# Gait variability following abrupt removal of external stabilization decreases with practice in incomplete spinal cord injury but increases in non-impaired individuals

**DOI:** 10.1186/s12984-018-0475-7

**Published:** 2019-01-07

**Authors:** Mengnan Mary Wu, Geoffrey L. Brown, Kwang-Youn A. Kim, Janis Kim, Keith E. Gordon

**Affiliations:** 1 Department of Physical Therapy and Human Movement Sciences, Feinberg School of Medicine, Northwestern University, Chicago, IL USA; 2 Department of Preventive Medicine (Biostatistics), Northwestern University, Chicago, IL USA; 3 Department of Kinesiology and Nutrition, University of Illinois Chicago, Chicago, IL USA; 4 Research Service, Edward Hines, Jr. VA Hospital, Hines, IL USA

**Keywords:** Gait, Locomotion, Spinal cord injury, Motor adaptation, Aftereffect, Savings, Slacking

## Abstract

**Background:**

Individuals with incomplete spinal cord injury (iSCI) exhibit considerable lateral center of mass (COM) movement variability during gait transitions from a stabilizing to unassisted environment, while non-impaired individuals do not. To understand how iSCI influences gait adaption, we examined persons with and without iSCI performing repeated locomotor transitions. We hypothesized that, with practice, individuals with iSCI would prioritize COM control performance during the transition as exhibited by a reduction in kinematic variability. In, contrast, we hypothesized that non-impaired individuals would prioritize control effort by decreasing muscular activity.

**Methods:**

Thirteen participants with iSCI and 12 non-impaired participants performed five treadmill-walking trials. During some trials, a cable-robot applied stabilizing lateral forces to the pelvis proportional in magnitude and opposite in direction to real-time lateral COM velocity. Each trial consisted of 300 continuous steps with or without a transition. During the first and last trials, no forces were applied and no transitions occurred (Null trials). During trials 2–4 (transition trials), the first 200 steps occurred in the stabilizing force field, forces were then abruptly removed, and 100 more unassisted steps were performed. We analyzed COM and step width variability, and hip abductor muscle activity during transitions (force removal until gait returned to steady state).

**Results:**

Participants with iSCI displayed large COM movement variability during the first transition but reduced variability with practice. During the first transition, lateral COM speed, lateral COM excursion, and step width were all more variable than during the first Null trial (*p* < 0.05). By the third transition, no metric was different from Null trials (*p* > 0.05).

In contrast, non-impaired participants’ movement variability during the first transition was not different from Null trials (*p* > 0.05). With practice, movement variability increased: lateral COM excursion was more variable during Transitions 2 and 3 versus the first Null trial (*p* < 0.05). Non-impaired participants decreased hip abductor activity from Transition 1 to 3 (*p* < 0.05).

**Conclusions:**

Individuals with iSCI demonstrated rapid motor savings. By the third transition, individuals with iSCI reduced locomotor variability to baseline levels. In contrast, non-impaired participants prioritized control effort over control performance. With practice transitioning, non-impaired participants increased locomotor variability and decreased muscular effort.

**Electronic supplementary material:**

The online version of this article (10.1186/s12984-018-0475-7) contains supplementary material, which is available to authorized users.

## Background

We have previously observed that when a laterally stabilizing external force field is abruptly removed during treadmill walking that individuals with and without incomplete spinal cord injury (iSCI) exhibit aftereffects of decreased step width that required 14 steps to return to steady state [1]. During this locomotor transition from a stabilizing environment to unassisted walking, individuals with iSCI also demonstrated a substantial increase in variability in center of mass (COM) speed and step width when compared to baseline performance [1]. For individuals with iSCI, this substantial locomotor variability during the transition may have posed a safety risk, and was thus likely a result of a temporary inability to control one’s mediolateral COM movement rather than a decision to not control variability. No change in locomotor variability during the transition was observed in non-impaired participants. This contrasting result suggests that in the absence of a wide base of support, non-impaired individuals were able to successfully utilize alternative methods to control mediolateral COM movement (e.g. potentially increased muscle co-activation).

The ability to rapidly and appropriately transition gait patterns to match changes in the external environment is an important skill that may improve with practice. Several studies have demonstrated savings – the phenomenon that previous learning speeds subsequent relearning – in a variety of motor tasks, including saccades [2], upper-limb reaching [3], and locomotion [4]. Several factors may enhance savings. In particular, abrupt changes in the external environment have been shown to result in more accurate perception and recall of the environment than gradual changes [4]. The positive effects of abrupt transitions on savings has been attributed to greater cognitive demand [5] and/or reliance on feedforward vs. feedback processes [4, 6]. Here we investigate the effects of practicing abrupt walking transitions from a highly stable environment to an unassisted environment in individuals with and without iSCI. We anticipate that the two groups will prioritize different control objectives during subsequent transitions based on the very different initial responses to this abrupt transition observed in our previous study [1].

We hypothesize that individuals with iSCI will reduce COM and step width variability with practice transitioning from a stabilizing environment to an unassisted environment. Reductions in variability with practice would suggest a prioritization of control performance. Such a prioritization can be observed in individuals with iSCI during steady-state walking conditions, where they exhibit persistent cautious gait patterns, including wide steps [7] and increased double-support time [8], that reflect a general strategy to improve their ability to resist and recover from perturbations [1]. These behaviors increase control performance but come with a significant associated metabolic [7, 9] and cognitive [10] cost.

In contrast, we hypothesize that non-impaired individuals will reduce muscle activity with practice transitioning from the stabilizing environment to an unassisted environment. As observed previously [1], the control methods used by non-impaired participants during a novel environment transition were successful for controlling mediolateral COM movement, but they may not have been efficient. Reductions in muscle activity with practice would suggest a prioritization of control effort. Franklin et al. have suggested a motor learning model in which the central nervous system first adapts control parameters to reduce error, and once errors are below a threshold, the nervous system will gradually reduce excessive muscle activity [11]. This motor “slacking” model in which people reduce control effort with practice has been observed in non-impaired individuals walking with a powered exoskeleton [12]. In accordance with Franklin’s model, we hypothesize that non-impaired individuals will prioritize reductions in control effort with practice because movement errors were substantially small during the locomotor transition from a stabilizing environment to unassisted walking [1, 11].

## Methods

### Participants

All participants gave written informed consent prior to beginning the study. Both the Northwestern University Institutional Review Board and the Edward Hines Jr. Veterans Administration Hospital Institutional Review Board approved the protocol. All participants were between 18 and 75 years of age. Inclusion criteria for participants with iSCI included: injury level between C1-T10, American Spinal Injury Association Impairment Scale (AIS) C or D [13], > 1 year post-injury, range of motion within functional limits of ambulation, and ability to walk 10 m without assistive devices. Participants with iSCI were excluded from the study if they: had excessive spasticity in the lower limbs, were unable to tolerate 45 min total of treadmill walking, had a history of recurrent fractures or known orthopedic problems in the lower extremity, or had concomitant central or peripheral neurologic injury. Non-impaired participants were excluded if they had any medical conditions limiting ambulatory ability. Participants did not alter medications for this study.

### Experimental setup

**Fig. 1:**
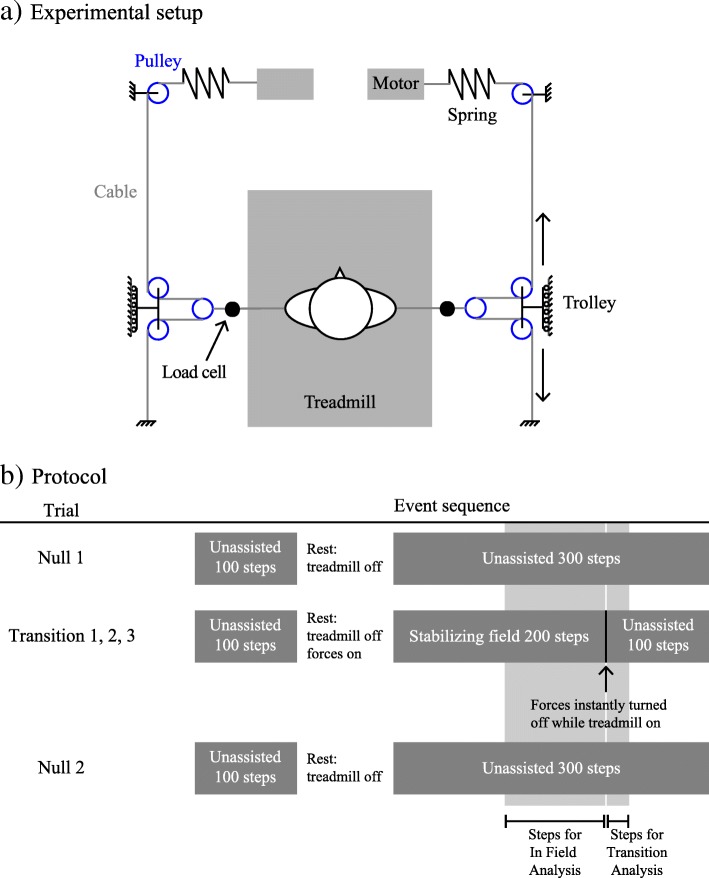
Experimental setup and protocol. **a** Experimental setup. Participants wore a trunk harness attached to a passive overhead safety support system and walked on an oversized treadmill. Lateral forces were created using a pair of series-elastic linear motors and transmitted via cables to a pelvic harness. Load cells measured the applied forces, which varied with experimental trials. Cables were routed through trollies that allowed participants free fore-aft motion. **b** Protocol. Participants performed five trials in the following order: Null 1 – unassisted walking with no transitions between environments; Transition 1, Transition 2, and Transition 3 - a stabilizing velocity-dependent lateral force field was applied to the participant and then abruptly removed; and Null 2 – unassisted walking with no transitions. During each trial, participants completed 400 steps at their preferred speed. The first 100 steps established baseline unassisted walking to control for fatigue or carryover. The treadmill was then stopped. For Null trials, the treadmill was simply restarted and unassisted walking continued for 300 steps to mimic the treadmill stopping/starting during the Transition trials. For the Transition trials, the following sequence of events occurred: a stabilizing force field was applied during standing, the treadmill was then restarted, participants walked 200 steps with stabilization, forces were instantly removed on-the-fly without stopping the treadmill, and participants walked unassisted for another 100 steps

All experiments took place in the Human Agility Laboratory at Northwestern University, Chicago, IL. Participants walked on an oversized treadmill – 1.39 m belt width (Tuff Tread, Willis, TX) – providing space to step laterally (Fig. 1a). Participants wore a trunk harness attached to a passive overhead safety support system (Aretech, Ashburn, VA) that provided no bodyweight support and allowed unrestricted travel across the treadmill.

During select walking periods, stabilizing lateral forces were applied to a pelvis harness using a pair of cables (Fig. 1a). Force on each cable was created by a series-elastic linear motor and measured by a load cell connected in series [14]. During periods when lateral forces were applied, participants experienced a variable lateral force proportional (50 Ns/m) in magnitude and opposite in direction to their real-time lateral COM velocity. This velocity-dependent resistance created by the applied force field reduced the self-generated lateral stabilization required to maintain straight-ahead walking. This velocity-dependent resistance is not a typical method used to provide stability in gait rehabilitation settings. Because the stabilization field is continuous and dependent on the participant’s motion, people can potentially form an internal model of the field and adjust movement patterns accordingly. A result of these properties is that people may demonstrate particularly strong aftereffects when the viscous stabilizing field is removed when compared to other forms of stabilization (e.g. handrails or manual assistance).

To measure kinematics, we placed 17 reflective markers on the pelvis and bilaterally on the greater trochanter, lateral knee, lateral malleolus, calcaneus, and 2nd and 5th metatarsals. An 11-camera motion capture system (Qualisys, Gothenburg Sweden) recorded 3D marker coordinates at 100 Hz. To measure EMG activity, we placed wireless surface electrodes (Delsys Trigno, Natick, MA) bilaterally on the gluteus medius (GM) hip abductor muscles since GM activity has been shown to significantly correlate with mediolateral foot placement [15]. EMG signals were recorded at 1000 Hz.

### Protocol

The study was a within-person design for each group (individuals with and without iSCI) since we expected significant variability in walking behavior between individuals. Thus, we examined how each participant changed relative to their baseline walking behavior.

First, to characterize the functional abilities of the study participants, we collected demographic information (all participants) and clinical outcome measures (only participants with iSCI) performed without assistive devices. The clinical outcome measures included: the lower extremity motor score portion of the International Standards for Neurological Classification of Spinal Cord Injury exam (ISNCSCI) [13], 10 Meter Walk Test [16] performed at maximum speed, Timed Up and Go [17], Walking Index for Spinal Cord Injury [18], and the Berg Balance Scale [19].

Next, we identified participants’ preferred treadmill walking speed (iSCI 0.58 ± 0.20 m/s; non-impaired 1.04 ± 0.20 m/s). Participants then practiced walking on the treadmill for 2 min at this speed to acclimate. Participants were instructed to walk as they felt most comfortable, swing their arms freely, and to allow their midline to oscillate laterally, with oscillations centered over a chalk line drawn along the mediolateral center of the treadmill belt. Participants were explicitly instructed not to try to walk on the chalk line like a tightrope. No handrails or other supports, including assistive devices, were available during walking. Participants with iSCI rested at least 2 min between trials, and all participants were given additional rest as requested.

Participants then performed five treadmill walking trials (Fig. 1b) in the following order:

- **Null 1** – unassisted walking with no transitions between environments;
- **Transition 1, Transition 2, and Transition 3** – participants walked with a stabilizing velocity-dependent lateral force field applied to their pelvis, the field was then abruptly removed and participants continued walking unassisted;
- **Null 2** – unassisted walking with no transitions.

Null trials were presented at the beginning and end of the experiment to monitor if baseline walking behavior changed during the course of the experiment. During each trial, participants completed 400 steps at their preferred speed. The first 100 steps was used to establish baseline unassisted walking (Null 1) and washout any potential carryover effects from previous trials (Transitions 1–3 and Null 2). The treadmill was then stopped. For Null trials, the treadmill was restarted and unassisted walking continued for 300 steps. For the Transition trials, the following sequence of events occurred: a stabilizing force field was applied during standing, the treadmill was then restarted, participants walked 200 steps in the stabilizing force field, forces were then removed on-the-fly without stopping the treadmill, and participants walked unassisted for another 100 steps.

To ensure that participants were aware of exactly when the transition from the stable environment to unassisted walking would occur, an auditory countdown of the last 5 steps before the stabilizing forces were shut off was provided during the Transition trials. During walking periods when no forces were applied, Null trials and unassisted walking during the Transition trials, the cables remained attached to the pelvic harness but hung without tension.

### Data post-processing

Kinematic marker data was processed using Visual3D (C-Motion, Germantown, MD) and a custom MATLAB (Mathworks, version 2016b, Natick, MA) program. Marker data was gap-filled and low-pass filtered (Butterworth, 6 Hz cut-off frequency). Time of initial foot contact (IC) and toe-off (TO) events were identified for each step based on fore-aft positions of the calcaneus and 5th metatarsal markers. Mediolateral COM position was calculated in Visual3D as the center of the “Visual3D pelvis model.”

To characterize control performance, we identified peak lateral COM Speed and calculated lateral COM Excursion and Step Width. COM velocity was calculated as the derivative of COM position. Peak lateral COM Speed was identified as the maximum absolute COM velocity between ipsilateral IC events, and lateral COM Excursion was calculated as the difference between the maximum and minimum lateral COM positions per stride. Step width was calculated as the mediolateral distance between the left and right 5th metatarsal markers at midstance.

To assess control effort and slacking behavior, we measured GM hip abductor EMG activity. EMG activity of the GM was analyzed during stance phase, when abductor activity is consistently highest [20]. EMG was preprocessed by removing the mean, clipping movement artifacts to four standard deviations of the mean, bandpassed with a sixth-order Butterworth filter at 20-400hz, and then low-passed at 50hz [15]. For each participant, we identified the maximum processed EMG activity during each of the first 100 steps of Null Trial 1 and took the average as the “mean peak EMG activity” per step during this period. We then normalized all processed EMG activity by dividing by the mean peak EMG activity. The stance phase RMS activity was then calculated per stride, and a mean over all the strides occurring during specific periods of interest (the transition period for Transition trials and corresponding time period for Null trials) was taken as the final metric. In several participants, we observed very large movement artifacts created by the interaction between the surface electrodes and the snug pelvic harness to which external forces were being directly applied. Although the use of low profile electrodes (Delsys Trigno Mini-Sensors) alleviated this issue for most participants, only EMG data from 9 non-impaired and 6 participants with iSCI were useable for analysis.

For the Transition trials, we identified the number of steps required for participants’ kinematics to return to steady state after the removal of the force field. These steps were later used to evaluate control performance during the transition between environments. To estimate the time course of the transition, we fit an exponential function to step width during the 100 steps following removal of the stabilizing force field. Our previous research found that this metric was most robust at describing the observed transition [1]. A Poisson general linear model found that participants with iSCI and non-impaired participants had significantly different time constants (9 steps for iSCI vs. 22 steps for non-impaired) for step width to return to steady state during Transition 1, even after controlling for age as a covariate (group: z = 4.858, *p* < 0.001, Additional file 1). Thus, we used each group’s mean time constant for all further analysis. This time constant represented the time length required for participants to transition their gait patterns between environments. Step width trends varied greatly over time and often non-exponentially during Transitions 2 and 3. Despite attempts with several algorithms, we were not able to fit a consistent model to the data for these trials. The algorithms were not robust in accounting for inter-subject and intra-subject variability. As such, we used the time constant identified during Transition 1 in order to compare all trials, with the implicit assumption that the time course of Transition 1 would be equal to or longer than Transitions 2 and 3 and that the same time period could be evaluated from the Null trials.

**Fig. 2:**
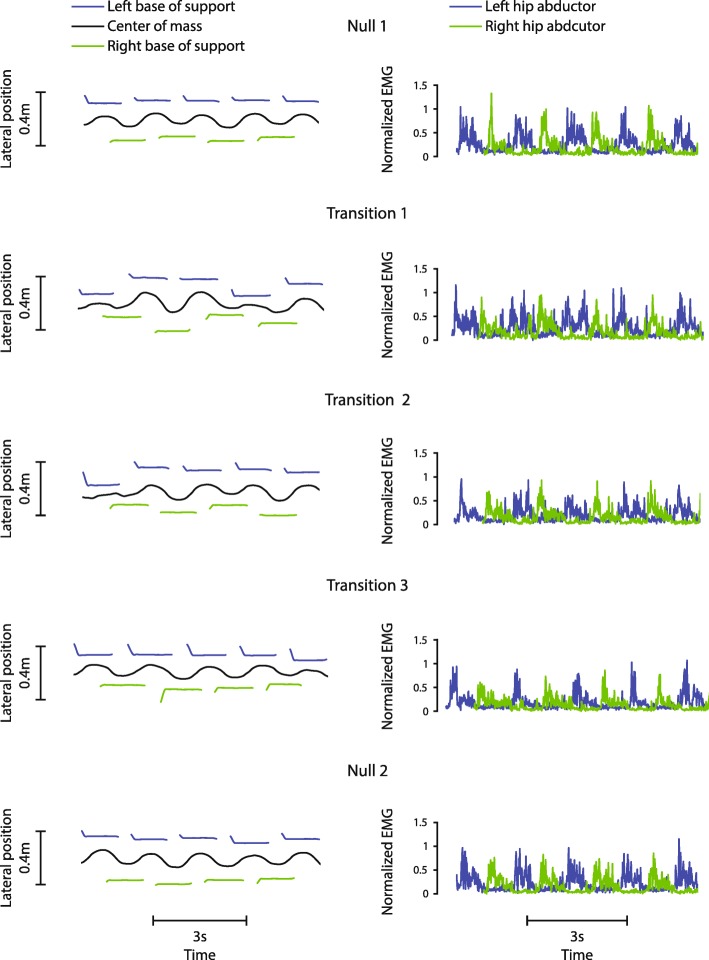
Example frontal plane kinematics and hip abductor EMG activity from a representative participant with iSCI. Frontal plane kinematics (left) and hip abductor EMG data (right) for a representative participant with iSCI during the first 9 steps of the second unassisted walking period of each trial. The group time constant of 9 steps represents the average number of steps across all participants with iSCI to return to steady state during Transition 1 and was used to calculate metrics for all trials for consistency. COM variability visibly decreased over repetitions of the transition, showing savings. Muscular activity showed a slight trend of decrease over repetitions of the transition

**Fig. 3:**
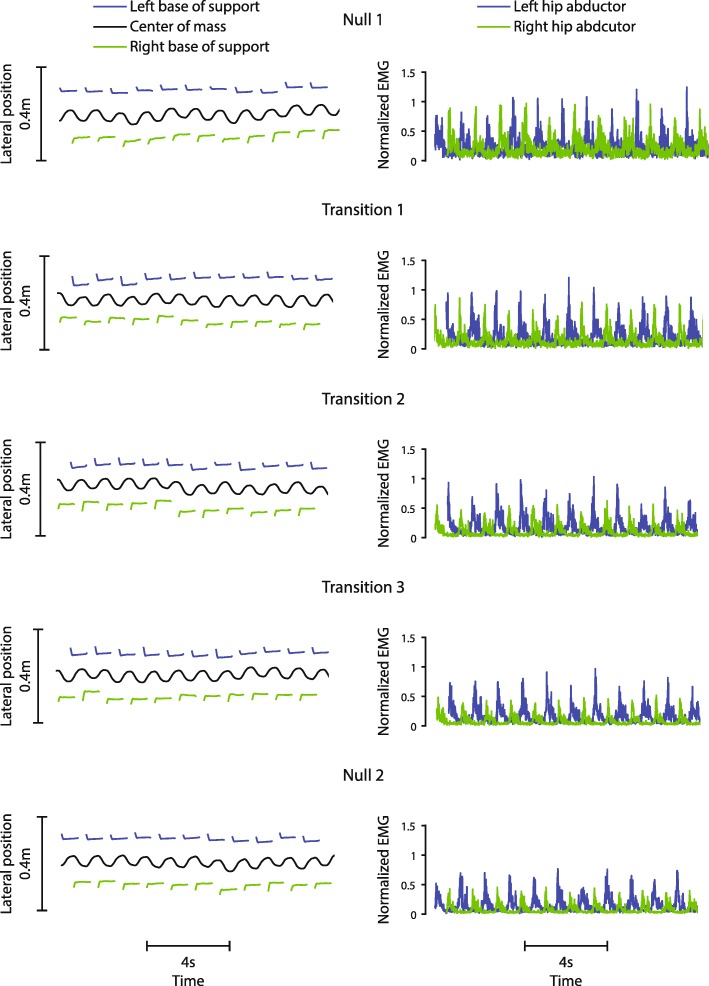
Example frontal plane kinematics and hip abductor EMG activity from a representative non-impaired participant. Frontal plane kinematics (left) and hip abductor EMG data (right) for a representative non-impaired participant during the first 22 steps of the second unassisted walking period of each trial. The group time constant of 22 steps represents the average number of steps across all non-impaired participants to return to steady state during Transition 1 and was used to calculate metrics for all trials for consistency. Muscular activity decreased over repetitions of the transition, showing slacking

Consequently, to assess gait during locomotor transitions between environments, we analyzed the *first* 9 steps of the last 100 steps of each trial for the iSCI group (Fig. 1b and example participant in Fig. 2) and the *first* 22 steps of the last 100 steps for the non-impaired group (Fig. 1b and example participant in Fig. 3). For Transition trials, the last 100 steps were unassisted and immediately followed walking in the stabilizing field. For Null trials, the last 100 steps were also unassisted but were part of a continuous period of unassisted walking. Although our primary interest was behavior during the locomotor transition, for completeness, we also analyzed the last 100 steps prior to the transition, which occurred either unassisted (Null trials) or in a stabilizing field (Transition trials).

### Statistical analysis

To investigate the change in walking metrics between trials (Null 1, Transition 1–3, and Null 2), we used separate one-way repeated measures ANOVAs for each group for the metrics: step width, peak lateral COM speed mean and variability, lateral COM excursion mean and variability, and mean hip EMG activity. For the repeated measures ANOVA, if sphericity was violated, the Greenhouse-Geisser (G-G) F-statistic and *p*-value were used to test the main effect. When a significant main effect was found, Bonferroni-corrected pairwise comparisons were made between trials. If assumptions of the parametric repeated measures ANOVA model were violated, Friedman’s test was used instead for main effects and Wilcoxon’s signed-rank test was used for pairwise comparisons. Significance was set at the *p* < 0.05 level for all tests and comparisons. This procedure was repeated to examine differences between trials both during the transition period and during walking in the force field.

## Results

### Participants

Thirteen ambulatory participants with chronic motor incomplete iSCI (all AIS D) and 12 non-impaired participants took part in the study. One participant with iSCI was excluded from analysis because he misinterpreted the instructions for the experimental protocol and stopped walking in the middle of the Transition 1 trial. Participants with iSCI were 56 ± 9 years, height 1.7 ± 0.1 m, weight 74.5 ± 16.9 kg, and 10 males/2 females (see Additional file 2 for clinical outcome measures). Non-impaired participants were 41 ± 15 years of age, height 1.6 ± 0.4 m, weight 83.5 kg, and 9 males/3 females.

### Environment transitions

**Fig. 4:**
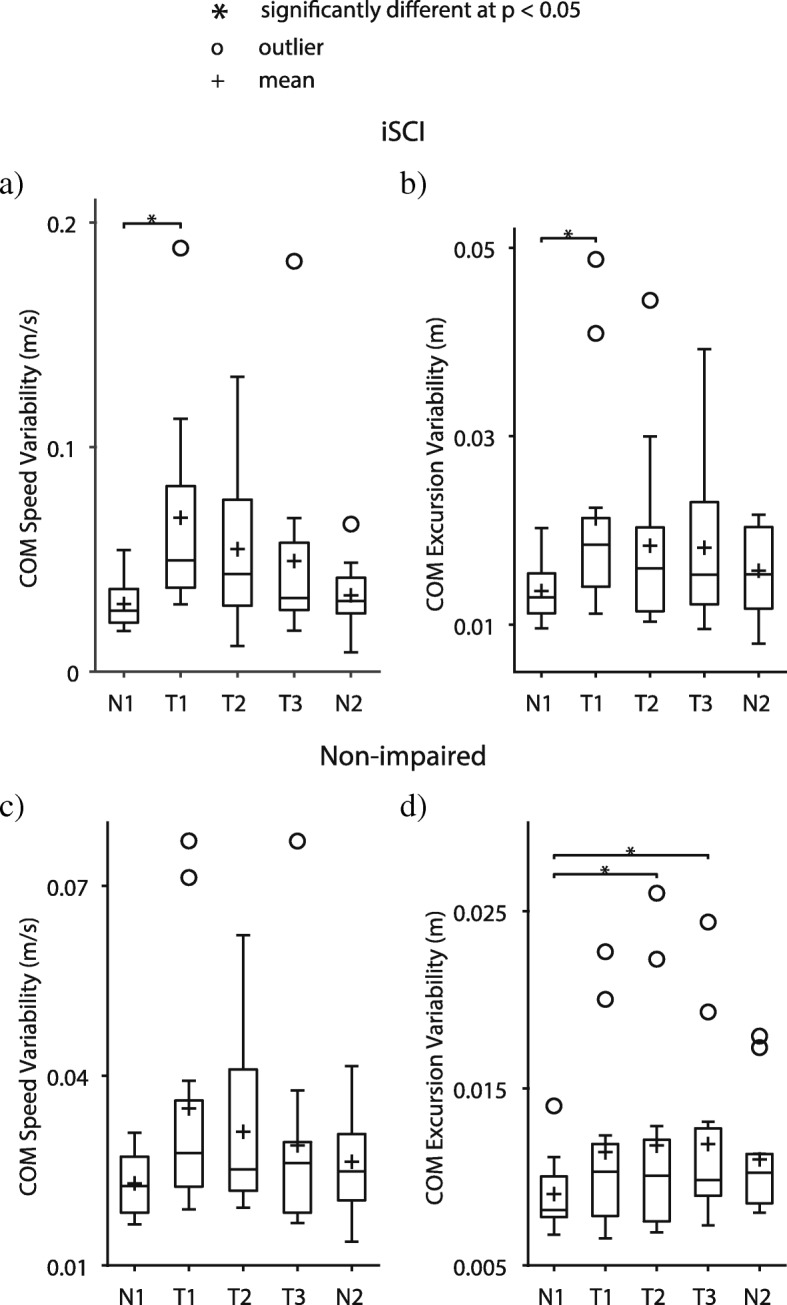
Mean and variability for Peak Lateral Center-of-mass Speed and Lateral Center-of-mass Excursion. Variability of peak lateral center-of-mass speed and lateral center-of-mass excursion during the transition period for Transition trials and corresponding time periods for Null trials. Participants with iSCI showed significantly more variability in (**a**) peak lateral center-of-mass (COM) speed per stride and (**b**) lateral COM excursion per stride during Transition 1 but decreased variability to Null trial levels with repetitions of transitions, demonstrating savings in control performance. Non-impaired participants did not change (**c**) lateral COM speed variability and significantly increased (**d**) variability of lateral COM excursion, showing worse control performance with repetitions of transitions. Trials: N1 = Null 1, T1 = Transition 1, T2 = Transition 2, T3 = Transition 3, N2 = Null 2

**Fig. 5:**
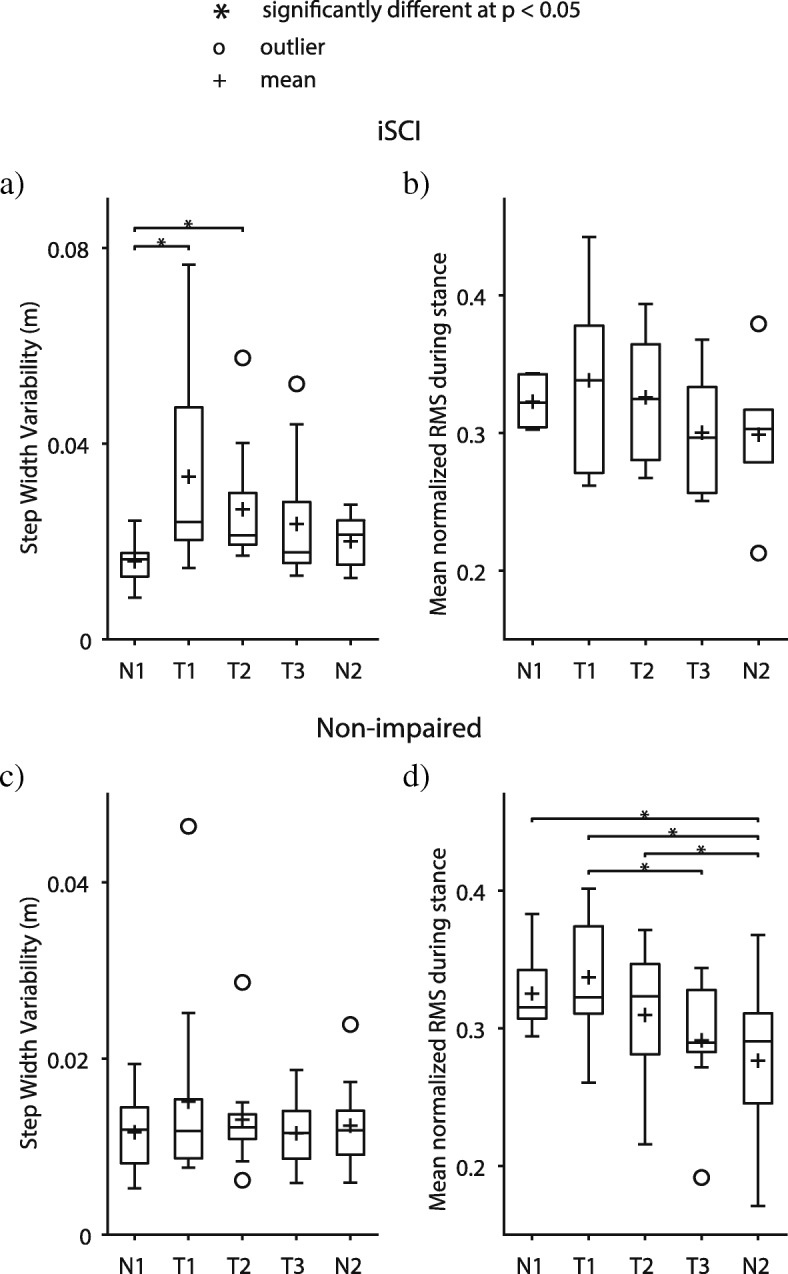
Step width variability and mean hip abductor EMG activity. Step width variability and mean hip abductor (Gluteus Medius) EMG activity during stance phase of the transition period for Transition trials and corresponding time periods for Null trials. **a** Participants with iSCI showed significantly higher step width variability during Transitions 1 and 2 and decreased variability to Null trial levels by Transition 3, demonstrating savings in control performance. **b** Participants with iSCI showed a non-significant trend of decreasing hip abductor activity with repetitions of transitions. **c** Non-impaired participants did not change step width variability with repetitions of transitions. **d** Non-impaired participants significantly decreased hip abductor activity with repetitions of transitions, exhibiting slacking. Trials: N1 = Null 1, T1 = Transition 1, T2 = Transition 2, T3 = Transition 3, N2 = Null 2

Consistent with our previous work [1], participants with iSCI exhibited large irregular lateral movements during the first transition from an externally stabilizing force field to unassisted walking (see video of example participant with iSCI in Additional file 4). Lateral COM speed variability (Fig. 4a), lateral COM excursion variability (Fig. 4b), and step width variability (Fig. 5a) significantly increased during Transition 1 vs. Null 1 (Table 1). However, by Transition 3, participants with iSCI adapted back to baseline walking behavior with no significant differences in any metric vs. Null trials (Table 1), showing evidence of savings. Hip abductor EMG activity showed no significant effects, but there was a trend for EMG activity to decrease with transition practice (Table 1 and Fig. 5b).

**Table 1:** Statistical analysis of the transition period of the unassisted period

Metric	Repeated Measures ANOVA				Friedman’s test			
	GG *p*-value	*p*-value	Significant pairs	*p*-value	Χ^2^	*p*-value	Significant pairs	*p*-value
iSCI								
COM speed mean		0.576						
COM speed variability					10.733	**0.030**	**N1 < T1**	**0.030**
COM excursion mean					8.267	0.082		
COM excursion variability					14.333	**0.006**	**N1 < T1**	**0.002**
Step width mean	0.11							
Step width variability					14.533	**0.006**	**N1 < T1**	**0.005**
							**N1 < T2**	**0.030**
Hip abductor activity		0.240						
Non-impaired								
COM speed mean		**0.019**	none					
COM speed variability					9.267	0.055		
COM excursion mean		0.468						
COM excursion variability					13.533	**0.009**	**N1 < T2**	**0.030**
							**N1 < T3**	**0.012**
Step width mean		**0.029**	none					
Step width variability					1.867	0.760		
Hip abductor activity		**< 0.0005**	**N1 > N2**	**0.048**				
			**T1 > T3**	**0.012**				
			**T1 > N2**	**0.005**				
			**T2 > N2**	**0.001**				

Greenhouse-Geisser (GG) correction was used when sphericity assumption was violated. Friedman’s nonparametric tests were used when normality assumptions were violated. Significant effects shown in **bold**. N1–2: Null 1 and 2 trials*;* T1–3: Transition 1–3 trials


**Additional file 4:** Video of a participant with iSCI walking in the stabilizing force field and during the transition period for Transition 1 and 3 trials and corresponding time period for Null 1 trial. (MP4 78462 kb)


In contrast, non-impaired participants did not appear visually unstable during Transition 1, and their COM movement variability *increased* significantly with transition practice. Specifically, COM excursion variability was significantly larger during Transitions 2 and 3 vs Null 1 (Table 1 and Fig. 4d). In addition, non-impaired participants significantly decreased hip abductor EMG activity from Transition 1 to 3 (Table 1 and Fig. 5d).

For safety reasons, research personnel provided physical support to participants on limited occasions when either the participant or researcher felt this was necessary to avoid a fall (Additional file 4 shows an example of the participant who received the most assistance). Examination of video footage showed that the participant in the sample video received 8 instances of support during Transition 1, two instances during Transition 2, and no support during Transition 3. Two other participants with iSCI each received a single instance of support. Non-impaired participants did not receive any support.

### Behavior in the force field

Comparisons of kinematics during the 100 steps in the stabilization field immediately prior to the transition in Transition trials vs. unassisted walking during the corresponding 100 steps in the Null trials (Fig. 1b) showed that both groups significantly reduced movement variability in the presence of the stabilizing force field (Additional file 3). Participants with iSCI significantly decreased means and variabilities for COM speed and excursion when walking in the stabilizing force field of all three Transition trials vs. unassisted walking of the Null trials (Additional file 3). Non-impaired participants significantly decreased mean COM speed and COM excursion mean and variability when walking in the stabilizing force field of all three Transition trials vs. the Null trials and significantly decreased step width during only the first Transition trial compared to the first Null trial (Additional file 3). Although both groups were affected by the presence of the stabilizing force field, variability in COM speed and excursion were not significantly different between the three stabilization trials.

Analysis of hip abductor activity suggests that slacking occurred in non-impaired participants. GM activity was similar during the Null trials and when walking in the stabilizing field during Transition trials 1 and 2. However, when walking in the stabilizing field during Transition trial 3, GM activity was statistically smaller than during the Null 1 trial (Additional file 3). There was a significant main effect of trial on hip abductor activity for participants with iSCI, but pairwise comparisons were not significant.

## Discussion

In support of our first hypothesis, with practice, all participants with iSCI, including participants who were highly challenged during Transition 1 (see Additional file 4), exhibited savings, demonstrating improved COM control performance during transitions between environments. Studies examining locomotor adaptation using a split-belt treadmill paradigm have also identified savings during transitions between walking environments [21, 22]. Several factors are thought to contribute to savings, including experience making abrupt changes between environments [4]. The savings observed in the current study may have been a result of error-based learning, which would have benefited from the relatively large movement errors [23] created by the abrupt changes in external stabilization. There is considerable evidence that the cerebellum plays an important role in the error-based learning that occurs with walking practice [24, 25]. Animal research also suggests that the lumber spinal cord in isolation can support locomotor adaptations to repeated external perturbations [26]. Participants in the current experiment had incomplete injuries, so it is possible that both spinal and supraspinal structures contributed to the observed improvements in locomotor transitions that occurred with practice.

In contrast to participants with iSCI, non-impaired participants did not show savings in control performance with repetitions of transitions. This is consistent with a previous experiment that also found no evidence of motor savings in non-impaired individuals following practice walking in a force field applied to the single limb [27]. The absence of savings in these experiments suggests that savings might only occur when people perceive sufficient challenge or risk. For learning to occur, it may not be enough that a kinematic error occurs, but rather that the central nervous system interprets the error as meaningful.

In support of our second hypothesis, non-impaired individuals adopted a “slacking” control strategy that reduces effort in response to repetitions of transitions from a stabilizing field to unassisted walking. This reduction in muscle activity during the transition was paralleled by decreased muscle activity *in* the stabilization force field with repeated exposures. Interestingly, the reduction in muscle activity with practice was accompanied by an unexpected increase in movement variability during the transition with practice. Franklin’s model of motor learning suggests that muscle activity should decrease only when movement errors are small [11]; movement variability levels likely remained subthreshold throughout all transition repetitions. That movement variability increased with practice may further suggest that non-impaired individuals were prioritizing control effort over control performance. This finding is consistent with past data suggesting that people can select more efficient walking patterns at the expense of stability [28, 29].

In addition, the greater time length of Transition 1 for non-impaired individuals (22 steps on average) vs. individuals with iSCI (9 steps on average) could also be explained by a greater tendency for non-impaired individuals to prioritize control effort at the expense of control performance (defined in terms of variability). While a more delayed reaction to a perturbation may result in less bounded behavior (less orbital stability), it may conserve energy and be beneficial to maintaining balance - the initial reaction to a perturbation may amplify the perturbation and a time delay may allow for more context-specific voluntary responses [30].

Hip abductor activity in participants with iSCI also showed a non-significant decreasing trend with repetitions of transitions. The lack of statistical significance could be attributed to the small number of participants with iSCI from whom clean EMG signals were collected, and, thus, this population may have also been trying to reduce control effort in parallel with efforts to improve control performance.

**Fig. 6:**
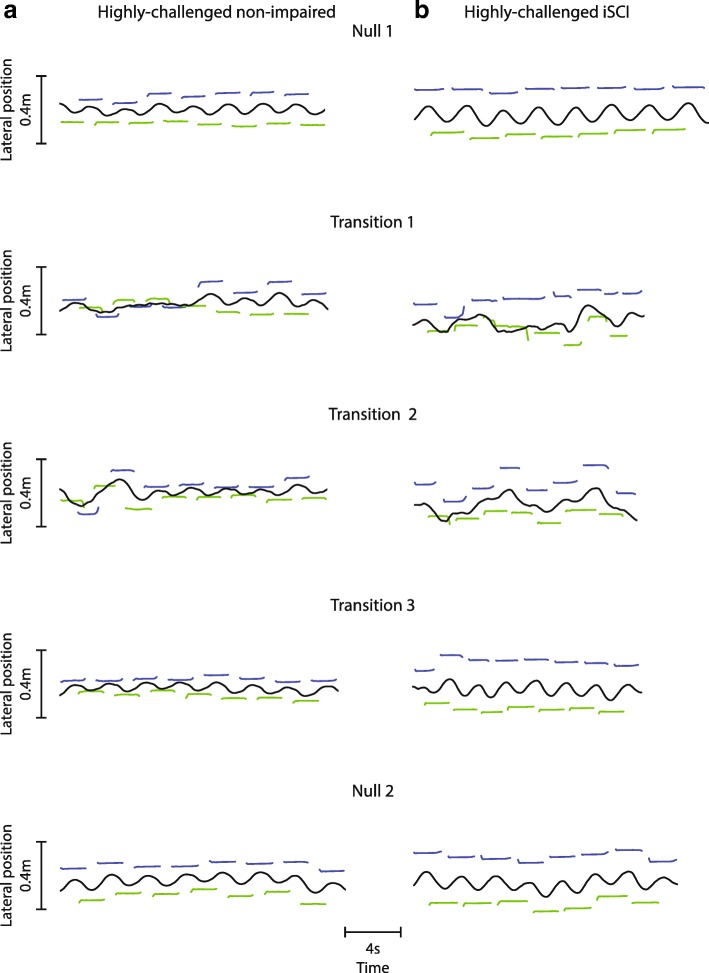
Frontal plane kinematics for highly-challenged participants. Frontal plane kinematics for (**a**) a highly-challenged non-impaired participant (left column) and (**b**) a highly-challenged participant with iSCI (right column) during the transition steps (the time constant for Transition 1 for all participants combined was 15 steps) for Transition trials and corresponding time periods for Null trials. The highly-challenged non-impaired participant maintained narrow step widths during Transitions 1–3 and exhibited significant variability in COM motion during Transitions 1 and 2. The highly-challenged participant with iSCI never reached steady state during the entire length (100 steps) of the unassisted period following removal of the stabilizing field. However, this individual showed savings in improved control of COM motion by Transition 3

Surprisingly, there were large differences in balance ability *within* each group, which were sometimes larger than the average differences *between* groups, which complicated our statistical analysis. For example, a highly-challenged non-impaired participant (Fig. 6a) maintained narrow step widths in all three transitions and exhibited significant variability in COM motion during the first two transitions. This multi-step persistence of behavior during Transition 3 was not observed in participants with iSCI (Figs. 2 and 6b). Further investigation is needed to identify if these within-group differences in balance ability can be predicted a priori. Given the large within-group differences and lack of age/gender matching, we did not directly compare the two groups statistically, but we did perform an additional analysis to examine the effect of age across all participants (iSCI and non-impaired). With all participants combined, we identified an average transition period of 15 steps during Transition 1 (using the same exponential fitting method described earlier) and found a significant correlation between age and step width variability during this period (*ρ* = 0.414, *p* < 0.05) (Additional file 5). The correlation suggests that age may be an important factor independent from spinal cord injury for identifying individuals who may experience challenges in walking transitions from stabilizing to unassisted environments.

There were several limitations in the current study. First, the method of using the transition length calculated from Transition 1 for all three transitions likely impacted the results, but was the most robust method for dealing with high inter-subject and inter-trial variability in step width data. The transition data was highly variable across trials, especially for Transition 2 and 3, where the participant likely learned from Transition 1 and explored strategies of responding to the transition. Based on visual inspection of data, we found it to be a fair assumption that the longest period of transition occurred during Transition 1, so we used that same length of data to analyze all subsequent transitions for consistency. Another factor contributing to difficulties in fitting a model to all three transitions was that the experimental protocol assumed that all participants would return to steady-state walking kinematics within the allotted 100 steps of the second unassisted period after removal of the stabilizing field. However, as shown in the video for the example participant with iSCI (Additional file 4), some participants had such a strong aftereffect that they did not reach steady state during this period. Another limitation was that some participants were provided brief instances of physical support from experimenters for safety. Physical support likely improved COM control performance and decreased control effort. Although limited, this physical support may have reduced our estimates of savings in participants with iSCI. A final limitation of this study relates to assumptions about control effort and kinematic variability. While we instructed participants to try and center their midline over a target location during walking, individuals’ interpretation of this task and level of motivation may have affected their effort to control variability. One way to address this issue in future studies would be to design more constrained and challenging walking tasks with clear measures of and feedback on error. Such tasks would help identify if changes in movement variability are directly related to changes in ability vs. preference in movement control.

## Conclusions

Ambulatory individuals with iSCI showed rapid and substantial improvements in their ability to reduce COM movement variability during a challenging transition from a stabilizing environment to unassisted walking. In contrast to participants with iSCI, non-impaired participants’ movement variability actually increased with practice, and was accompanied by reductions in muscle activation. The strategy observed in non-impaired participants possibly reflected a trade-off in control performance for lower control effort. Thus, control priorities of different populations may influence what aspect of performance change with practice of a locomotor balance task.

## Additional files

Additional file 1: Time course of transition from stabilization to unassisted walking during Transition 1. Description: Figure of step width vs. step data and exponential fits to find time constant for step width to return to steady state for a) example participant with iSCI and b) example non-impaired participant, and c) total data for each group. (PDF 861 kb)

Additional file 2: Table of demographic and clinical outcome measures for participants with iSCI. (XLSX 12 kb)

Additional file 3: Table of statistical analysis results for the last 100 steps in the null or stabilizing force field prior to the transition for both participant groups. (XLSX 14 kb)

Additional file 5: Step width variability vs. age during Transition 1. Description: Figure of step width variability data for all participants (with iSCI and non-impaired), linear fit to combined data, Pearson’s correlation, and *p*-value of significance of correlation. (PDF 79 kb)

## Abbreviations

COM: Center-of-mass
GM: Gluteus Medius
iSCI: Incomplete spinal cord injury
