# Supplementary material for: Gait variability following abrupt removal of external stabilization decreases with practice in incomplete spinal cord injury but increases in non-impaired individuals
Source: J Neuroeng Rehabil. 2019 Jan 7;16:4. doi: 10.1186/s12984-018-0475-7 (PMC6322313; doi:10.1186/s12984-018-0475-7)
Supplement: Supplementary file 2 — Time course of transition from stabilization to unassisted walking during Transition 1. Description: Figure of step width vs. step data and exponential fits to find time constant for step width to return to steady state for a) example participant with iSCI and b) example non-impaired participant, and c) total data for each group. (PDF 861 kb) [file 12984_2018_475_MOESM1_ESM.pdf]

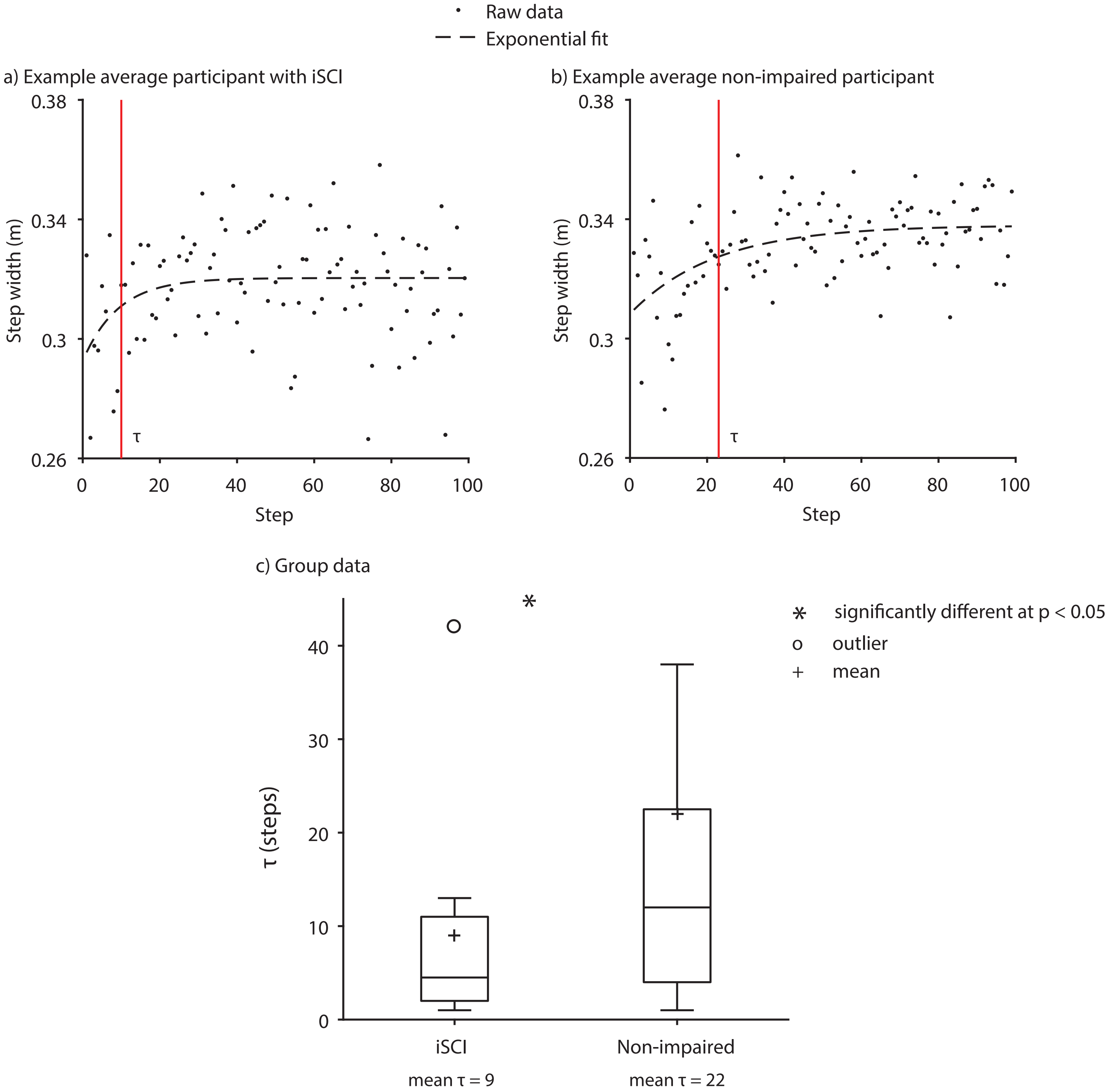

Raw data
Exponential fit
a) Example average participant with iSCI
b) Example average non-impaired participant
0.38
0.34
0.3
0.26
Step width (m)
τ
0
20
40
60
80
100
Step
c) Group data
significantly different at p < 0.05
outlier
mean
τ (steps)
40
30
20
10
0
iSCI
Non-impaired
mean τ = 9
mean τ = 22
