# Supplementary figures and images for: Gait variability following abrupt removal of external stabilization decreases with practice in incomplete spinal cord injury but increases in non-impaired individuals
Source: J Neuroeng Rehabil. 2019 Jan 7;16:4. doi: 10.1186/s12984-018-0475-7 (PMC6322313; doi:10.1186/s12984-018-0475-7)

- × iSCI  
○ Non-impaired  
— Linear fit all subjects

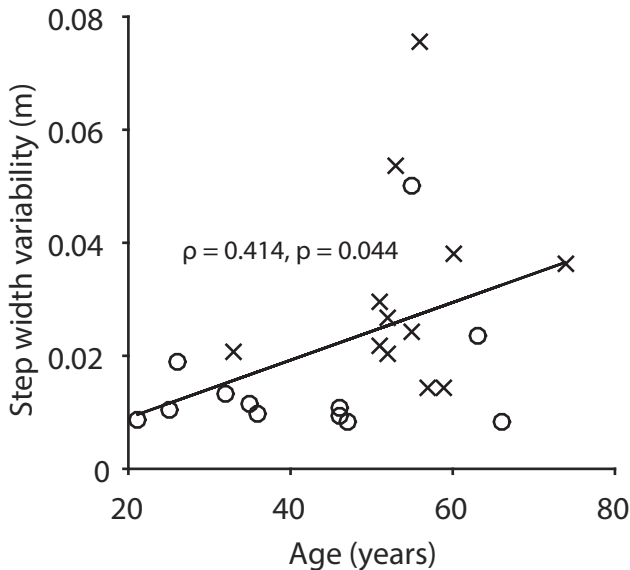

Supplement: Supplementary file 5 — Step width variability vs. age during Transition 1. Description: Figure of step width variability data for all participants (with iSCI and non-impaired), linear fit to combined data, Pearson’s correlation, and p-value of significance of correlation. (PDF 79 kb) [file 12984_2018_475_MOESM5_ESM.pdf]
